# A human *in vitro* 3D neo-cartilage model to explore the response of OA risk genes to hyper-physiological mechanical stress

**DOI:** 10.1016/j.ocarto.2021.100231

**Published:** 2021-12-25

**Authors:** Ritchie G.M. Timmermans, Niek G.C. Bloks, Margo Tuerlings, Marcella van Hoolwerff, Rob G.H.H. Nelissen, Robert J.P. van der Wal, Peter M. van der Kraan, Arjen B. Blom, Martijn H.J. van den Bosch, Yolande F.M. Ramos, Ingrid Meulenbelt

**Affiliations:** a Department of Biomedical Data Sciences, Section Molecular Epidemiology, Leiden University Medical Centre, Leiden, the Netherlands; b Experimental Rheumatology, Radboud University Medical Centre, Nijmegen, the Netherlands; c Department of Orthopaedics, Leiden University Medical Centre, Leiden, the Netherlands

**Keywords:** Mechanical stress, Human neo-cartilage, Osteoarthritis, OA risk genes

## Abstract

**Objective:**

Due to the complexity and heterogeneity of osteoarthritis (OA) pathophysiology, studying the interaction between intrinsic molecular changes in chondrocytes after hyper-physiological mechanical stress (MS) and aberrant signalling of OA risk genes remains a challenge. In this study we set out to set up an *in vitro* 3D neo cartilage pellet model that enables us to explore the responses of OA risk genes to hyper-physiological MS.

**Design:**

Human primary chondrocyte neo-cartilage pellets were exposed for 2 days to 2 ​× ​10 ​min of hyper-physiological dynamic MS attained by a 20% strain and a frequency of 5 ​Hz. In order to assess cartilage damage, sulphated glycosaminoglycan (sGAG) content in the neo-cartilage was quantified using Alcian blue staining and a dimethyl methylene blue (DMMB) assay, while cleavage of aggrecan was visualized by immunohistochemical staining of aggrecan neo-epitope NITEGE. In addition, changes in expression levels of catabolic, anabolic and hypertrophic genes, and of three OA risk genes; *IL11*, *MGP* and *TGFA* were determined.

**Results:**

Hyper-physiological MS induced cartilage damage, as reflected by decreased sGAG content. mRNA levels of aggrecanase *ADAMTS5* were increased, while hypertrophic gene RUNX2 was downregulated. MS increased expression of pro-apoptotic marker *NOXA*. Furthermore, 20% MS led to increased expression of all three OA risk genes *IL11*, *MGP* and *TGFA*.

**Conclusions:**

We established a human *in vitro* model in which hyper-physiological MS induced cartilage damage and catabolic signalling. Next, we demonstrated its usage to study OA risk genes and their response to the mechanical aspects of OA pathophysiology.

## 1 Introduction

Although osteoarthritis (OA) is considered a whole joint disease, a major hallmark of OA pathophysiology is progressive degradation of articular cartilage. Articular cartilage, with the chondrocyte being its unique resident cell, is a highly specialized tissue that is laid down during development and assures buffering of mechanical force from articular joints throughout life [1]. Nonetheless, its vulnerability to OA is highlighted by the poor regenerative capacity of chondrocytes e.g. micro traumas that arise after hyper-physiological mechanical stress. As such, hyper-physiological mechanical stress (MS) on articular cartilage is considered one of the primary factors that triggers onset of OA [2].

To identify inherent etiologic factors of OA onset, various comprehensive genome-wide genetic association studies were performed resulting in the identification of multiple strong OA risk genes such as interleukin 11 *(IL11),* transforming growth factor alpha (*TGFA)* and matrix Gla protein (*MGP)*. The functions of these OA genes, suggest that deviations in cartilage maintenance processes are major pathways underlying OA pathology in humans [[3], [4], [5]].

To study intrinsic molecular changes in chondrocytes after hyper-physiological load in interaction with aberrant function of OA risk genes, a human *in vitro* model is required that incorporates functional cartilage tissue units and the ability to apply MS, as an important environmental cue to triggering OA-like changes [6]. Such a model would thus contribute to study intrinsic noxious molecular responses of chondrocytes as well as modifying roles of strong OA risk genes towards efficient risk prediction. Proper understanding of these molecular responses of chondrocytes to MS, could aid in the development of an effective evidence-based framework for OA therapies, and advice on healthy physical activity with and without disease.

To this end, we have exploited a 3D *in vitro* cartilage model of primary human chondrocytes that deposit high quality physiological cartilage [7] and induced cartilage damage by hyper-physiological loading. We determined the effects of hyper-physiologic dynamic MS on the extracellular matrix (ECM) and expression of catabolic, anabolic, hypertrophic and apoptotic genes over time. Moreover, we applied the model to gain more insight into how MS affects known OA risk genes, such as *IL11*, *MGP* and *TGFA*.

## 2 Methods

### 2.1 Cell culture and mechanical loading of articular neo-cartilage

Human primary chondrocytes were isolated from joints of patients undergoing total joint replacement due to end-stage OA, included in the RAAK study. 3D neo-cartilage pellets were formed using 2.5 ​× ​10^5^ chondrocytes and cultured for 11 days in chondrogenic medium. For two consecutive days, the neo-cartilage was subjected to MS (2 ​× ​10 ​min) with 20% compression and a frequency of 5 ​Hz. For additional details, see online supplementary methods.

### 2.2 Quantification of gene expression

Per donor, RNA from two replicate pellets were pooled and reverse transcribed into cDNA. Gene expression was analysed using RT-qPCR. For additional details, see online supplementary methods.

### 2.3 Histological analysis and immunohistochemistry

Neo-cartilage samples were fixed in 4% formaldehyde and embedded in paraffin. Sections were stained with Alcian Blue and Nuclear Fast Red, or immunohistochemically stained for the aggrecanase-induced neo-epitope NITEGE or IL-11. Relative pixel intensity was determined using ImageJ-Fiji v1.52. For additional details, see online supplementary methods.

### 2.4 Dimethyl methylene blue assay (DMMB) for glycosaminoglycan quantification in neo-cartilage

Sulphated glycosaminoglycan (sGAG) concentrations in the pellets were measured using the Dimethyl Methylene Blue assay. For additional details, see online supplementary methods.

### 2.5 Statistical analysis

All graphs were created using Graphpad Prism 8.0.2 and statistics were performed in IBM SPSS Statistics 25. *P*-values <0.05 were considered significant. For additional details, see online supplementary methods.

## 3 Results

### 3.1 *Dynamic hyper-physiological MS in human neo-cartilage increases sGAG release and upregulates ADAMTS5* expression

**Fig. 1:**
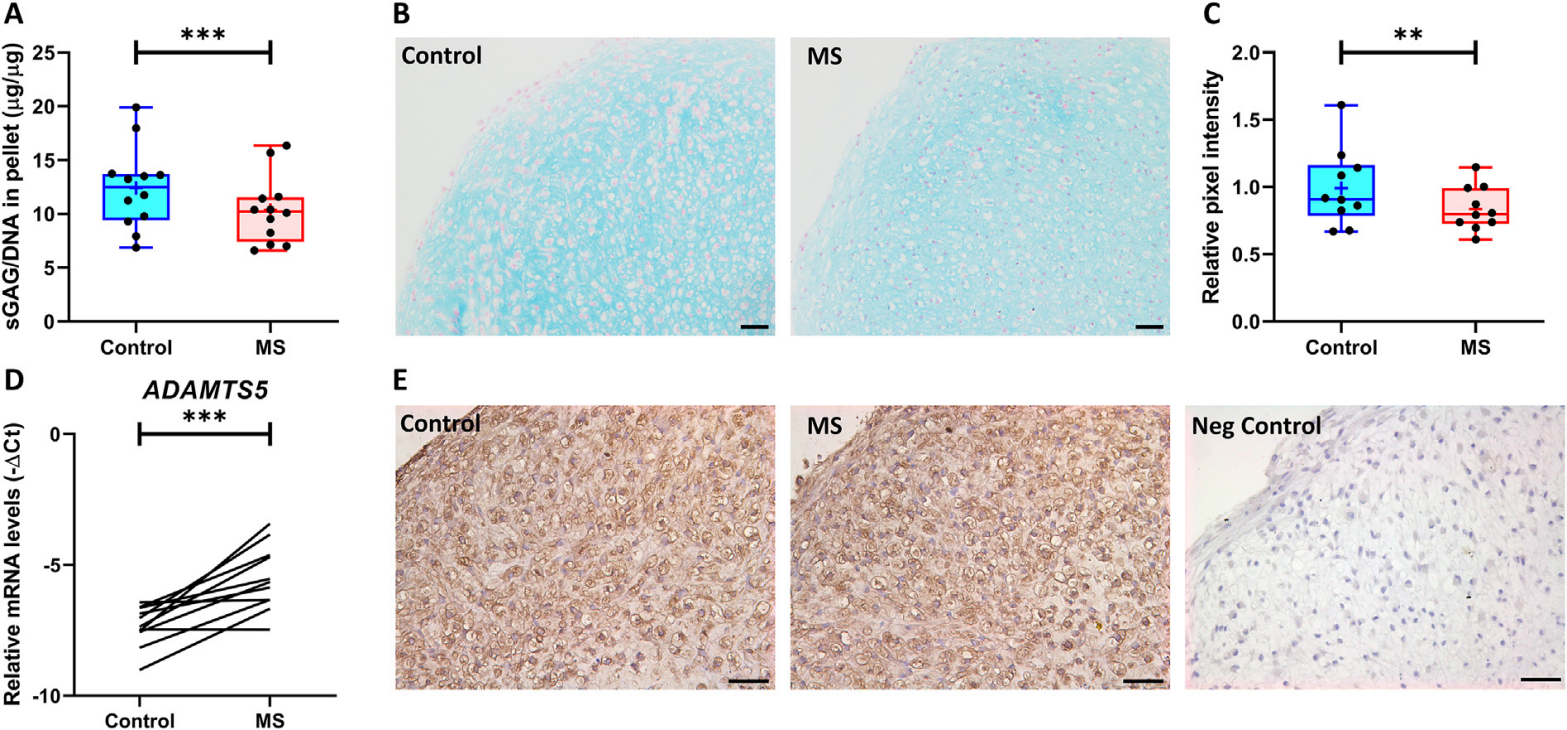
**Assessment of cartilage damage in the neo-cartilage after two days of exposure to 20% MS**. (**A**) sGAG content in the neo-cartilage as determined by DMMB, corrected for DNA content. (**B**) Representative images of Alcian Blue staining and (**C**) subsequent quantification of intensities calculated using Fiji-ImageJ. (**D**) RT-qPCR analysis of catabolic marker *ADAMTS5*. (**E**) Representative images of neo-cartilage stained for aggrecan neo-epitope NITEGE, scale bar ​= ​50 ​μm sGAG content and Alcian Blue intensities are presented in a boxplot depicting the median, lower and upper quartiles. Mean is depicted as ​+ ​and each dot represents a single donor. Lines represent differences in *ADAMTS5* mRNA expression levels between control and 20% MS within a single donor. *P*-values of mean differences in sGAG levels (*n* ​= ​12 donors) and Alcian Blue intensities (*n* ​= ​10 donors) between control and 20% MS were estimated by generalized estimating equations. *P*-values of mean differences in *ADAMTS5* mRNA expression were estimated using a paired samples T-test (*n* ​= ​11 donors). ∗∗∗*P* ​< ​0.001.

The mean ​± ​SD age of the donors used in this model was 65 ​± ​10 years with 11 females and 1 male (Table S2). Prior to exposing the neo-cartilage to 20% MS for two consecutive days, we explored the effects of a single 20% MS. As shown in Fig. S1, we did not observe any significant changes in Alcian Blue intensity, sGAG content and release after the first 20% MS. Therefore, we subjected the neo-cartilage for two days to 20% MS to increase cartilage damage. As shown in Fig. 1, 20% MS significantly decreased sGAG content (20% MS: 10.37 ​± ​1.77 ​μg/μg versus Control: 12.40 ​± ​2.17 ​μg/μg, *P* ​< ​3.3 ​× ​10^−19^, Fig. 1A), which was accompanied by a significant reduction in Alcian Blue intensity (FC ​= ​0.82 ​± ​0.08, *P* ​< ​8.0 ​× ​10^−3^, Fig. 1B and C). However, we did not observe any differences in sGAG concentrations in the medium (Fig S1B). On mRNA level, we measured a consistent increase in *ADAMTS5* expression (FC ​= ​5.02 ​± ​3.16, *P* ​= ​6.0 ​× ​10^−4^, Fig. 1D and Table S4). Notably, we observed downregulation of hypertrophic marker *RUNX2* (FC ​= ​0.64 ​± ​0.3, *P* ​= ​3.6 ​× ​10^−2^, Table S4). In contrast, *MMP3*, *MMP13* and *COL10A1* expression did not significantly change (Table S4). Furthermore, we observed an upregulation of pro-apoptotic gene *NOXA* (FC ​= ​2.46 ​± ​0.67, *P* ​= ​8.5 ​× ​10^−5^, Table S4). To investigate whether the increased *ADAMTS5* mRNA expression led to aggrecan degradation in the matrix of the neo-cartilage, we performed immunohistochemical staining on the aggrecanase-induced aggrecan neo-epitope NITEGE. As shown in Fig. 1E, we showed positive NITEGE staining in the neo-cartilage, localized in the ECM and intracellularly, but were not able to observe differences in expression between control and 20% MS.

### 3.2 20% MS induces expression of OA risk genes

**Fig. 2:**
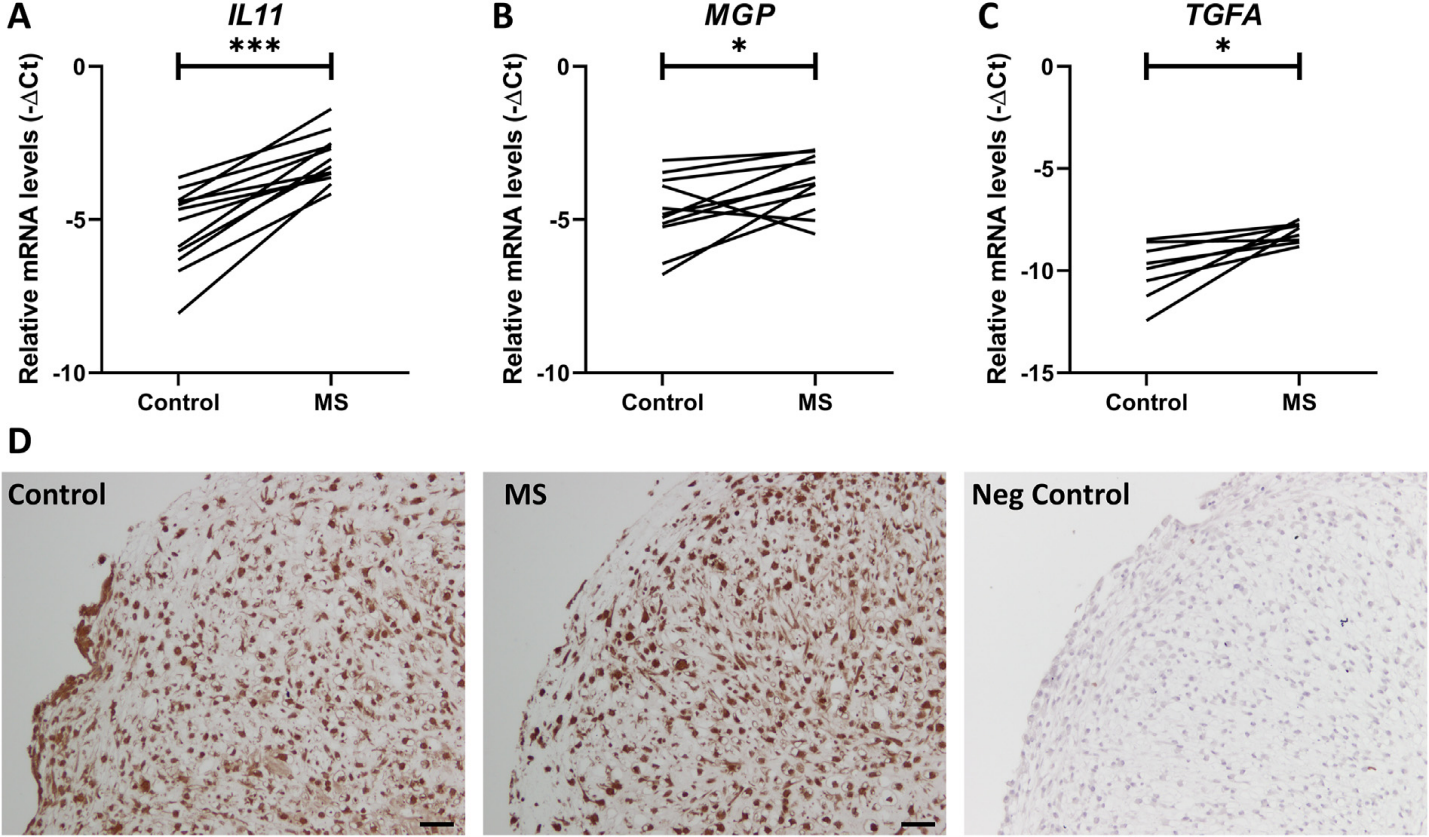
**Gene expression levels of OA risk genes *IL11*, *MGP* and *TGFA* and IL-11 protein expression in the neo-cartilage after two days of exposure to 20% MS**. RT-qPCR analysis of (**A**) *IL11*, (**B**) *MGP* and (**C**) *TGFA*. Lines represent differences in mRNA expression levels between control and MS in individual donors. (**D**) Representative images of neo-cartilage stained for IL-11, scale bar ​= ​50 ​μm. *P*-values of mean differences in mRNA expression (*n* ​≥ ​8 donors) were estimated using a paired samples T-test. ∗*P* ​< ​0.05, ∗∗∗*P* ​< ​0.001.

To explore the behaviour of known OA risk genes in neo-cartilage upon 20% MS, we determined expression of three known OA risk genes, *IL11*, *MGP* and *TGFA*. These risk genes confer their risk to OA either by subtle increase in gene expression levels during OA, or by alterations in the amino acid sequence of their respective protein, thereby affecting signalling of downstream targets (Table S1). As shown in Fig. 2, all three genes, *IL11* (FC ​= ​6.28 ​± ​3.12, *P* ​= ​1.2 ​× ​10^−5^; Fig. 2A), *MGP* (FC ​= ​2.52 ​± ​1.37, *P* ​= ​3.3 ​× ​10^−2^; Fig. 2B) and *TGFA* (FC ​= ​6.27 ​± ​6.61, *P* ​= ​1.1 ​× ​10^−2^; Fig. 2C) showed a strong consistent increase in mRNA levels compared to their control. Next, we assessed protein levels of the highest upregulated OA risk gene, *IL11*, in the neo-cartilage. While IL-11 was expressed in the neo-cartilage, no differences were detected between control and 20% MS (Fig. 2D).

## 4 Discussion

In this study, we present a human *in vitro* neo-cartilage pellet model to study behaviour of OA risk genes after MS-induced cartilage damage. We applied injurious hyper-physiologic dynamic MS to the neo-cartilage that led to catabolic responses by means of upregulation of *ADAMTS5* gene expression, increased apoptosis, and early cartilage damage as a result of proteoglycan loss. Furthermore, the model was then successfully used to confirm that several known OA risk genes showed similar expression patterns in response to MS as what is observed in OA articular cartilage [8].

The loading magnitudes of the MS and the microenvironment of the chondrocytes play a key role in the type of response chondrocytes will have [9]. To initiate a pathophysiological response, we chose dynamical 20% strain (2 ​× ​10 ​min) at 5 ​Hz for 2 consecutive days, that was marked by strong significant increase in *ADAMTS5* expression concomitant with loss of sGAGs in the neo-cartilage and increased apoptosis, but without consistent changes in *MMP13* (Table S4). This is different to the recently demonstrated effects in long-term 65% mechanically stressed human osteochondral explants that showed a strong increase in *MMP13* expression, but no response of *ADAMTS5* [10]. This shows that the catabolic responses to mechanical stress strongly depend on the strain, time and type of model being used. Nonetheless, we did not detect any changes in sGAG release. This is possibly due to that during the MS, the neo-cartilage was transferred from the medium to saline and transferred back to the medium afterwards. It is most likely that the observed proteoglycan loss in the neo-cartilage occurred directly during the MS, and was therefore not measurable in the medium. Furthermore, we were not able to measure differences in expression of the aggrecan neo-epitope NITEGE, which might be due to the possibility that the observed *ADAMTS5* upregulation was not yet visible on a protein level.

As cartilage tissue is usually collected during arthroplasty surgery for end stage of OA, the effect of excessive MS on healthy joints (i.e. cartilage) remains unknown. Therefore, researchers turn to human *ex vivo* and *in vitro* models to study the early effects of excessive mechanical loading on chondrocytes and their microenvironment. Over time, various load-based models of OA have been introduced, including monolayer, 3D-and explant cultures, which all differ in chondrocyte state, matrix composition and their response to MS [11]. In our study, chondrocytes were seeded in high density pellets, they deposit their own ECM that resembles the native cartilage on matrix composition [7]. Moreover, we have previously demonstrated that this neo-cartilage is highly similar to the autologous cartilage on the epigenetic level, which are crucial mechanisms to dynamically regulate gene expression in articular chondrocytes upon stress and disease [12]. Furthermore, the primary chondrocytes in this study were collected from macroscopically preserved areas of OA joints. As these cells are in a state between healthy and lesioned OA chondrocytes, they are therefore suitable to study the processes that initiate the transition towards OA. The findings of this study have to be seen in light of some limitations. Since this model is a single cell type model, the interaction between cartilage and underlying bone is lacking. It is known that the subchondral bone plays an important role in the responses on mechanical stress in the articular joint. In contrast to articular cartilage, the neo-cartilage exists of high density seeded chondrocytes, and together with the orientation of the formed collagen fibres, may influence the response on MS. While damage occurred as a result of MS, we did not observe any changes in expression of anabolic genes *COL2A1* and *ACAN*. This might be due to timing or, since the chondrocytes in the neocartilage are continuously producing ECM components, endogenous expression levels are high, which may overrule the effects of the applied MS. The direction of effect of *ADAMTS5* expression, sGAG content and Alcian Blue were consistent across almost all donors. However, the fold changes, likely due to donor heterogeneity, is subject to variation and hence inaccurate. Larger sample sizes are required to increase the accuracy of the fold change.

An important part of this study was to explore the responses of three OA risk genes after MS and compare this to what is observed in the articular cartilage of OA patients. *IL11*, *MGP*, and *TGFA* showed significant upregulation upon 20% MS. Similarly, these genes are upregulated in macroscopically lesioned compared to preserved OA cartilage, confirming their role in cartilage maintenance processes, with *IL11* showing the strongest effect in both lesioned OA cartilage and after MS in the current model [8]. While the downstream effects of IL-11 in MS-induced cartilage damage are still unclear, an important first step is to determine the effects of IL-11 on catabolic and anabolic activity in our model. Styrkarsdottir et al. [5] showed an association between a missense mutation in the *IL11* gene, leading to an unstable protein, and higher odds ratios of hip OA. Furthermore, during OA, expression of the IL-11 receptor alpha (*IL1*1RA) is downregulated [8]. Taken together, we postulate that during OA, IL-11 might have protective effects, but may be unable to properly exert its function. *MGP* has previously been identified as a strong OA risk gene [4]. As a regulator of extracellular calcium levels, low levels of MGP result in higher calcification of the articular cartilage and reduced bone mineral density. While *MGP* is upregulated during OA, the risk allele rs1800801-T is associated with lower expression of *MGP* in articular cartilage [13].

In conclusion, we established an *in vitro* model that mimics hyper-physiological dynamic MS-induced cartilage damage in human neo-cartilage, paired with upregulation of *ADAMTS5*. We propose this model as a suitable platform for high throughput screening of potential drugs targeted against *ADAMTS5*. Furthermore, we demonstrated that this model can be used to study the behaviour of OA risk genes and how they may connect to the mechanical aspects of OA pathophysiology.

## Contributions

Study concept and design: Ritchie G.M. Timmermans, Niek G.C. Bloks, Peter M. van der Kraan, Martijn H. J. van den Bosch, Arjen B. Blom, Yolande F.M. Ramos and Ingrid Meulenbelt. Acquisition of material and data: Ritchie G.M. Timmermans, Niek G.C. Bloks, Margo Tuerlings, Marcella van Hoolwerff, Robert J.P. van der Wal, Rob G.H.H. Nelissen. Data analysis: Ritchie G.M. Timmermans, Niek G.C. Bloks, Yolande. F.M. Ramos and Ingrid Meulenbelt. Preparation of the manuscript: Ritchie G.M. Timmermans, Niek G.C. Bloks, Yolande. F.M. Ramos and Ingrid Meulenbelt. Critical reviewing and approval of the manuscript: All authors.

## Role of the funding source

Data is generated within the scope of the Medical Delta programs Regenerative Medicine 4D: Generating complex tissues with stem cells and printing technology and Improving Mobility with Technology. Furthermore, the research leading to these results has received funding from the 10.13039/100018286Dutch Arthritis Society (DAF-17-1-401) and the 10.13039/100000002National Institutes of Health (AG15768 and AG46927).

## Compliance with ethics guidelines

The RAAK study has been approved by the medical ethical committee of the Leiden University Medical Center (P08.239/P19.013). Written informed consent was obtained from all patients, and patients had the right to withdraw at any time.

## Authorship

All authors should have made substantial contributions to all of the following: (1) the conception and design of the study, or acquisition of data, or analysis and interpretation of data, (2) drafting the article or revising it critically for important intellectual content, (3) final approval of the version to be submitted. By signing below each author also verifies that he (she) confirms that neither this manuscript, nor one with substantially similar content, has been submitted, accepted or published elsewhere (except as an abstract). Each manuscript must be accompanied by a declaration of contributions relating to sections (1), (2) and (3) above. This declaration should also name one or more authors who take responsibility for the integrity of the work as a whole, from inception to finished article. These declarations will be included in the published manuscript.

## Acknowledgement of other contributors

All contributors who do not meet the criteria for authorship as defined above should be listed in an acknowledgements section. Examples of those who might be acknowledged include a person who provided purely technical help, writing assistance, or a department chair who provided only general support. Such contributors must give their consent to being named. Authors should disclose whether they had any writing assistance and identify the entity that paid for this assistance.

## Conflict of interest

At the end of the text, under a subheading “Conflict of interest statement” all authors must disclose any financial and personal relationships with other people or organisations that could inappropriately influence (bias) their work. Examples of potential conflicts of interest include employment, consultancies, stock ownership, honoraria, paid expert testimony, patent applications/registrations, and research grants or other funding.

## Declaration of funding

All sources of funding should be declared as an acknowledgement at the end of the text.

## Role of the funding source

Authors should declare the role of study sponsors, if any, in the study design, in the collection, analysis and interpretation of data; in the writing of the manuscript; and in the decision to submit the manuscript for publication. If the study sponsors had no such involvement, the authors should state this.

## Studies involving humans or animals

Clinical trials or other experimentation on humans must be in accordance with the ethical standards of the responsible committee on human experimentation (institutional and national) *and* with the Helsinki Declaration of 1975, as revised in 2000. Randomized controlled trials should follow the Consolidated Standards of Reporting Trials (CONSORT) guidelines and be registered in a public trials registry.

Studies involving experiments with animals were in accordance with institution guidelines.

Please sign below to certify your manuscript complies with the above requirements and then upload this form at https://www.editorialmanager.com/oac/

## Declaration of competing interest

All authors declare that they have nothing to disclose.

## Appendix A Supplementary data

Multimedia component 2 Multimedia component 2

figs1Fig. S1 **Assessment of damage in the neo-cartilage after the first day of 20% MS.** (**A-B**) Representative images of Alcian Blue staining and quantification of Alcian Blue intensity calculated using Fiji-ImageJ. Scale bar = 50 μm. (**C**) sGAG content in the neo-cartilage as determined by DMMB. (**D**) sGAG concentrations in the medium after the first and the second day of MS as determined by DMMB. Data are presented in a boxplot depicting the median, lower and upper quartiles. Mean is depicted as +, each dot represents a single donor, and ∗ represents a statistical outlier. *P*-values of mean differences in Alcian Blue intensities (N = 10 donors) and sGAG levels (N = 17 donors) between controls and MS neo-cartilage were estimated by generalized estimating equations (GEE). Fig. S1
