# Supplementary material for: A human in vitro 3D neo-cartilage model to explore the response of OA risk genes to hyper-physiological mechanical stress
Source: Osteoarthr Cartil Open. 2021 Dec 25;4(1):100231. doi: 10.1016/j.ocarto.2021.100231 (PMC9718246; doi:10.1016/j.ocarto.2021.100231)
Supplement: Multimedia component 1 [file mmc1.docx]

### **Supplemental Methods**

#### **Cell culture and mechanical loading of articular neo-cartilage**

The effects of hyper-physiological MS were investigated using our previously described human 3D *in vitro* neo-cartilage [1]. In short, chondrocytes were isolated from preserved areas of joints of patients undergoing total joint replacement due to end-stage OA included in the Research in Articular Osteoarthritis Cartilage (RAAK) study [2]. The age range of the patients was 52-68 years, with an average (SD) age of 65 (10) years, and mostly female (92%, **Table S2**). Cells were expanded for two passages and pellets (250,000 cells/pellet) were created by centrifugation. The following day, pellets were incubated in chondrogenic medium to induce chondrogenesis. Within 11 days, the chondrocytes have deposited high quality neo-cartilage and were subjected to MS using a Mach-1 mechanical testing system (Biomomentum, Laval, Canada). MS was applied twice for 10 minutes, with a frequency of 5 Hz, a strain of 20% and a recovery interval of 10 minutes at two consecutive days. Samples were collected 12 hours after MS.

#### **RT-qPCR**

Per donor, two replicate neo-cartilage pellets were collected in TRIzol (Invitrogen™, Carlsbad, CA, USA) and RNA was isolated using the RNeasy Mini Kit (Qiagen, Venlo, the Netherlands) according to manufacturer’s protocol. DNA contamination was removed by treating the RNA with RNase-Free DNase (Qiagen, Venlo, the Netherlands). RNA quality (A260/280: 1.7-2.0) was assessed using the Nanodrop. RNA concentrations were measured with the Qubit® 2.0 Fluorometer (Invitrogen™, Carlsbad, CA, USA) using the RNA HS Assay Kit (Invitrogen™, Carlsbad, CA, USA)., respectively, with an A260/280 between 1.7-2.0. RNA was reverse transcribed into cDNA using the Transcriptor First Strand cDNA Synthesis Kit (Roche, Basel, Switzerland). cDNA was amplified using FastStart SYBR Green Master (Roche, Basel, Switzerland) and mRNA expression was measured in triplicates in a MicroAmp™ Optical 384-Well Reaction Plate (ThermoFisher Scientific, Landsmeer, the Netherlands), using the QuantStudio™ Flex Real-Time PCR system (Applied Biosystems™, Foster City, CA, USA), with the following cycling conditions: 10 min 95 °C; 10 sec 95 °C, 30 sec 60 °C, 20 sec 72 °C (45 cycles); 1 min 65 °C and 15 sec 95 °C . Primer efficiency was tested using a cDNA dilution series, and primers were considered efficient with an efficiency between 90% and 110%. -ΔCt expression levels were calculated using two housekeeping genes *GAPDH* and *SDHA*, with the following formula: *ΔCt = Ct (gene of interest) – Ct (average housekeeping genes)*. Both housekeeping genes were stably expressed in this model. Fold changes were calculated using the 2^-ΔΔCt^ method with *ΔΔCt = ΔCt (MS) – ΔCt (Control)*. Primer sequences are listed in **Table S3**.

#### **Histological analysis and immunohistochemistry**

Neo-cartilage samples were fixed in 4% formaldehyde and embedded in paraffin. Sections were stained with Alcian Blue (Sigma-Aldrich, Zwijndrecht, the Netherlands) and Nuclear Fast Red (Sigma-Aldrich, Zwijndrecht, the Netherlands). Microscopic images were loaded into ImageJ-Fiji v1.52. Images were split into three colour channels, selecting the channel containing the Alcian Blue staining. Images were corrected for uneven illumination using the rolling ball algorithm. The average pixel intensity was measured, corrected for background, and relative pixel intensities were calculated using the control as reference group. Aggrecanase activity and localization of IL-11 in the neo-cartilage was visualized immunohistochemically. Sections were stained for the aggrecanase-induced neo-epitope NITEGE as described previously [3]. In short, antigen retrieval was done by treating deparaffinized sections with hyaluronidase (10 mg/ml, Sigma, Zwijndrecht, the Netherlands) and a citrate buffer (10 mM, pH 6.0). Sections were incubated overnight with a primary antibody raised against the NITEGE neo-epitope (5 µg/ml), a kind gift from John Mort (McGill University, Montreal, Canada), or a rabbit IgG control (5 µg/ml, Agilent, Santa Clara, CA, USA), followed by incubation with VECTASTAIN® Elite ABC-HRP Kit (Vector Laboratories, Burlingame, CA, USA). For IL-11, antigen retrieval was done by treating deparaffinized sections with proteinase K (5 µg/ml, Qiagen, Venlo, The Netherlands) and hyaluronidase (5 mg/ml, Sigma, Zwijndrecht, the Netherlands). Sections were incubated overnight with a primary antibody raised against human IL-11 (1:100, ThermoFisher Scientific, Landsmeer, the Netherlands), followed by incubation with a HRP conjugated secondary antibody (ImmunoLogic, Duiven, the Netherlands). Peroxidase binding for both IL-11 and NITEGE was visualized using diaminobenzidine, and sections were counterstained with haematoxylin.

#### **Dimethyl Methylene Blue Assay (DMMB) for glycosaminoglycan quantification in neo-cartilage**

Sulphated glycosaminoglycan (sGAG) concentrations in the neo-cartilage (µg sGAG/µg DNA) and in the medium (µg SGAG/ml medium) was measured using the Farndale Dimethyl Methylene Blue (DMMB, Sigma, Zwijndrecht, the Netherlands) method [4]. Chondroitin sulphate (Sigma, Zwijndrecht, the Netherlands) was used as a reference standard. Absorbance was measured at 535 and 595 using a microplate reader (Synergy HT, Biotek, Winooski, VT, USA). Neo-cartilage sGAG concentrations were corrected for DNA content measured with the Qubit® 2.0 Fluorometer (Invitrogen™, Carlsbad, CA, USA) using the dsDNA HS Assay Kit (Invitrogen™, Carlsbad, CA, USA).

#### **Statistical analysis**

All graphs were created using GraphPad Prism 8.0.2. -ΔCt line graphs were created to show the direction of effect of mRNA levels from individual donors. All data was tested for normal distribution using the Shapiro-Wilk test. Statistical differences in -ΔCt expression levels were analysed using a paired *t*-test. Statistical significance of mean differences in Alcian Blue intensities, neo-cartilage and medium sGAG levels were estimated using a generalized estimating equation (GEE). Since perfect pairs were absent, by applying a GEE, we could adjust for dependencies among donors by adding a random effect for sample donors. We followed a linear GEE model, with Alcian Blue intensities and sGAG levels as dependent variable and condition as a factor. Statistics were performed in IBM SPSS Statistics 25. *P*-values < 0.05 were considered significant.

***References***

1. Bomer N, den Hollander W, Ramos YFM, Bos SD, van der Breggen R, Lakenberg N, et al. Underlying molecular mechanisms of DIO2 susceptibility in symptomatic osteoarthritis. Annals of the Rheumatic Diseases. 2015;74(8):1571. <https://doi.org/10.1136/annrheumdis-2013-204739>.

2. Ramos YF, den Hollander W, Bovée JV, Bomer N, van der Breggen R, Lakenberg N, et al. Genes involved in the osteoarthritis process identified through genome wide expression analysis in articular cartilage; the RAAK study. PLoS One. 2014;9(7):e103056. <https://doi.org/10.1371/journal.pone.0103056>.

3. van den Bosch MH, Blom AB, Kram V, Maeda A, Sikka S, Gabet Y, et al. WISP1/CCN4 aggravates cartilage degeneration in experimental osteoarthritis. Osteoarthritis and Cartilage. 2017;25(11):1900-11. <https://doi.org/10.1016/j.joca.2017.07.012>.

4. Farndale RW, Sayers CA, Barrett AJ. A Direct Spectrophotometric Microassay for Sulfated Glycosaminoglycans in Cartilage Cultures. Connective Tissue Research. 1982;9(4):247-8. <https://doi.org/10.3109/03008208209160269>.
