# Supplementary material for: A human in vitro 3D neo-cartilage model to explore the response of OA risk genes to hyper-physiological mechanical stress
Source: Osteoarthr Cartil Open. 2021 Dec 25;4(1):100231. doi: 10.1016/j.ocarto.2021.100231 (PMC9718246; doi:10.1016/j.ocarto.2021.100231)
Supplement: Multimedia component 2 [file mmc2.docx]

#### **Supplementary Table S1 – SNPs conferring risk to OA, and their associated genes, location, amino acid change, phenotype, effector allele (EA), minor allele frequency (MAF), odds ratio and effect.**

| **SNP** | **Gene** | **Location** | **Amino-acid change** | **Phenotype** | **EA** | **MAF** | **Odds Ratio** | **Expression in OA articular cartilage** | **Effect** | **References** |
| --- | --- | --- | --- | --- | --- | --- | --- | --- | --- | --- |
| rs4252548 | *IL11* | Exonic | p.Arg112His | Hip OA | T | 0.01 | 1.3 (1.2-1.4) | FC = 22.8, *P* = 1,5x10^-20^ | Reduced thermal stability | [1-3] |
| rs4764133 | *MGP* | Intergenic | - | Hand OA | T | 0.28 | 1.09 (1.04-1.13) | FC = 1.44, *P* = 2.1x10^-2^ | In high LD with rs1800801 (R^2^ = 0.95) and rs4236 (R^2^ = 0.86) | [1, 4, 5] |
|  |  |  |  | Finger OA |  |  | 1.12 (1.07-1.17) |  |  |  |
|  |  |  |  | Thumb OA |  |  | 1.07 (1.03-1.12) |  |  |  |
| rs1800801 | *MGP* | Intergenic | - | - | T | 0.26 | - | FC = 1.44, *P* = 2.1x10^-2^ | Allelic imbalance in OA | [1, 5] |
| rs4236 | *MGP* | Exonic | p.Thr127Ala | Hip OA | C | 0.39 | - | FC = 1.44, *P* = 2.1x10^-2^ | Allelic imbalance in OA | [1, 5] |
| rs2862851 | *TGFA* | Intronic | - | Hip and knee OA | T | 0.39 | 1.41 | FC = 1.50, *P* = 1.3x10^-3^ | - | [1, 6, 7] |

**Supplementary Table S2 – Patient characteristics**

| **Characteristic** | **One day 20% MS (N=17)** | **Two days 20% MS (N=12)** |
| --- | --- | --- |
| **Sex-no (%)** |  |  |
| Female | 15 (88%) | 11 (92%) |
| Male | 2 (12%) | 1 (8%) |
| **Age-yr** |  |  |
| Mean (SD) | 67 (9) | 65 (10) |
| Range | 52-86 | 52-86 |

**Supplementary Table S3 – Primer sequences**

| **Gene (primer)** | **Forward sequence** | **Reverse sequence** |
| --- | --- | --- |
| *GAPDH* | 5'-TGCCATGTAGACCCCTTGAAG-3' | 5'-ATGGTACATGACAAGGTGCGG-3' |
| *SDHA* | 5'-TGGAGCTGCAGAACCTGATG-3' | 5'-TGTAGTCTTCCCTGGCATGC-3' |
| *MMP3* | 5'-GAGGCATCCACACCCTAGGTT-3' | 5'-TCAGAAATGGCTGCATCGATT-3' |
| *MMP13* | 5'-TTGAGCTGGACTCATTGTCG-3' | 5'-GGAGCCTCTCAGTCATGGAG-3' |
| *ADAMTS5* | 5'-TGGCTCACGAAATCGGACAT-3' | 5'-GCGCTTATCTTCTGTGGAACC-3' |
| *COL2A1* | 5'-CTACCCCAATCCAGCAAACGT-3' | 5'-AGGTGATGTTCTGGGAGCCTT-3' |
| *ACAN* | 5'-AGAGACTCACACAGTCGAAACAGC-3' | 5'-CTATGTTACAGTGCTCGCCAGTG-3' |
| *SOX9* | 5'-CCCCAACAGATCGCCTACAG-3' | 5'-CTGGAGTTCTGGTGGTCGGT-3' |
| *RUNX2* | 5'-TGTGGTTACTGTCATGGCG-3' | 5'-AGGTAGCTACTTGGGGAGGA-3' |
| *COL10A1* | 5'-GGCAACAGCATTATGACCCA-3' | 5'-TGAGATCGATGATGGCACTCC-3' |
| *IL11* | 5'-CTCTACAGCTCCCAGGTGTGC-3' | 5'-AGGTAGGACAGTAGGTCCGCT-3' |
| *MGP* | 5'-CGCCCCCAGATTGATAAGTA-3' | 5'-TCTCCTTTGACCCTCACTGC-3' |
| *TGFA* | 5'-GCAGGTTTTTGGTGCAGGAG-3' | 5'-GGTGATGGCCTGCTTCTTCT-3' |
| *NOXA* | 5'-GGAGATGCCTGGGAAGAAG-3' | 5'-CCTGAGTTGAGTAGCACACTCG-3' |
| *BAK1* | 5’-GGGGACGACATCAACCGACGC-3’ | 5’-CGAAGCGGGTCACCTGGCCTA-3’ |
| *CASP7* | 5’-GCTGACTTCCTCTTCGCCTA-3’ | 5’-CAAACCAGGAGCCTCTTCCT-3’ |
| *BIRC5 (*Survivin*)* | 5'-AGCCCTTTCTCAAGGACCA-3' | 5'-CAGCTCCTTGAAGCAGAAGAA-3' |

#### **Supplementary Table S4 – Gene expression in neo-cartilage pellets after one and two days of 20% MS**. RT-qPCR analysis of catabolic markers MMP3, MMP13 and ADAMTS5, anabolic markers COL2A1, ACAN and SOX9, hypertrophic markers RUNX2 and COL10A1, and apoptotic genes NOXA and SURVIVIN, comparing one day of 20% MS (n=17 donors) and two days of 20% MS (n=11 donors) to their respective controls. Fold changes were calculated using the 2^-ΔΔCt^_­_ method. Statistical differences between –ΔCt values of control and MS groups were determined using a paired sample T-test.

|  | **One day 20% MS** | | | | | | | |  | **Two days 20% MS** | | | | | | | |
| --- | --- | --- | --- | --- | --- | --- | --- | --- | --- | --- | --- | --- | --- | --- | --- | --- | --- |
| **Gene** | **Fold Change** | **FC 95% CI** | | **Mean** | **Std. Error Mean** | **95% CI** | | ***P*-value** |  | **Fold Change** | **FC 95% CI** | | **Mean** | **Std. Error Mean** | **95% CI** | | ***P*-value** |
|  |  | **Lower** | **Upper** |  |  | **Lower** | **Upper** |  |  |  | **Lower** | **Upper** |  |  | **Lower** | **Upper** |  |
| **Catabolic** |  |  |  |  |  |  |  |  |  |  |  |  |  |  |  |  |  |
| *MMP3* | 2.05 | 1.23 | 2.88 | 0.70 | 0.24 | 0.18 | 1.22 | **1.1x10^-2^** |  | 1.23 | 0.71 | 1.75 | 0.08 | 0.22 | -0.41 | 0.57 | 7.2x10^-1^ |
| *MMP13* | 1.15 | 0.72 | 1.58 | -0.21 | 0.31 | -0.87 | 0.45 | 5.1x10^-1^ |  | 0.78 | 0.50 | 1.07 | -0.61 | 0.28 | -1.23 | 0.01 | 5.4x10^-2^ |
| *ADAMTS5* | 4.18 | 2.60 | 5.75 | 1.77 | 0.26 | 1.20 | 2.33 | **1.4x10^-5^** |  | 5.02 | 1.86 | 8.18 | 1.84 | 0.38 | 1.00 | 2.68 | **6.0x10^-4^** |
| **Anabolic** |  |  |  |  |  |  |  |  |  |  |  |  |  |  |  |  |  |
| *COL2A1* | 0.95 | 0.58 | 1.32 | -0.57 | 0.35 | -1.31 | 0.17 | 1.2x10^-1^ |  | 0.95 | 0.49 | 1.41 | -0.43 | 0.36 | -1.23 | 0.38 | 2.6x10^-1^ |
| *ACAN* | 1.14 | 0.51 | 1.76 | -0.67 | 0.42 | -1.57 | 0.23 | 1.3x10^-1^ |  | 1.56 | 0.26 | 2.86 | -0.56 | 0.65 | -2.01 | 0.89 | 4.1x10^-1^ |
| *SOX9* | 1.15 | 0.34 | 1.96 | -0.47 | 0.46 | -1.50 | 0.56 | 3.4x10^-1^ |  | 2.66 | 0.60 | 4.72 | 0.80 | 0.53 | -0.46 | 2.07 | 1.8x10^-1^ |
| **Hypertrophic** |  |  |  |  |  |  |  |  |  |  |  |  |  |  |  |  |  |
| *RUNX2* | 1.05 | 0.41 | 1.69 | -0.36 | 0.35 | -1.15 | 0.43 | 3.3x10^-1^ |  | 0.64 | 0.34 | 0.94 | -0.75 | 0.26 | -1.43 | -0.07 | **3.6x10^-2^** |
| *COL10A1* | 1.12 | 0.41 | 1.83 | -0.51 | 0.40 | -1.38 | 0.36 | 2.2x10^-1^ |  | 1.38 | 0.70 | 2.06 | 0.30 | 0.28 | -0.39 | 0.99 | 3.3x10^-1^ |
| ***Pro-*apoptotic** |  |  |  |  |  |  |  |  |  |  |  |  |  |  |  |  |  |
| *NOXA* | - | - | - | - | - | - | - | - |  | 2.50 | 1.90 | 3.10 | 1.21 | 0.19 | 0.78 | 1.64 | **8.5x10^-5^** |
| *BAK1* | - | - | - | - | - | - | - | - |  | 1.63 | 0.30 | 2.96 | 0.90 | 0.62 | -0.49 | 2.29 | 1.8x10^-1^ |
| *CASP7* | - | - | - | - | - | - | - | - |  | 1.59 | 1.01 | 2.17 | 0.45 | 0.26 | -0.14 | 1.04 | 1.2x10^-1^ |
| ***Anti-apoptotic*** |  |  |  |  |  |  |  |  |  |  |  |  |  |  |  |  |  |
| *BIRC5 (Survivin)* | - | - | - | - | - | - | - | - |  | 1.76 | 0.19 | 3.33 | -0.12 | 0.59 | -1,43 | 1.18 | 8.3x10^-1^ |

#### **Supplementary Table S5 – Gene expression in neo-cartilage pellets after the two days of 20% MS**. RT-qPCR analysis of IL11, MGP and TGFA comparing 20% MS to their respective controls (n≥8 donors). Fold changes were calculated using the 2^-ΔΔCt^_­_ method. Statistical differences between –ΔCt values of control and MS groups were determined using a paired sample T-test.

|  | **20% MS** | | | | | | | |
| --- | --- | --- | --- | --- | --- | --- | --- | --- |
| **Gene** | **Fold Change** | **FC 95% CI** | | **Mean** | **Std. Error Mean** | **95% CI** | | **P-value** |
|  |  | **Lower** | **Upper** |  |  | **Lower** | **Upper** |  |
| *IL11* | 6.28 | 3.16 | 9.40 | 2.29 | 0.30 | 1.62 | 2.96 | **1.2x10^-5^** |
| *MGP* | 2.52 | 1.15 | 3.89 | 0.91 | 0.37 | 0.09 | 1.73 | **3.3x10^-2^** |
| *TGFA* | 6.27 | -0.34 | 12.88 | 1.84 | 0.54 | 0.57 | 3.12 | **1.1x10^-2^** |

## **References**

1. Coutinho de Almeida R, Ramos YFM, Mahfouz A, den Hollander W, Lakenberg N, Houtman E, et al. RNA sequencing data integration reveals an miRNA interactome of osteoarthritis cartilage. Ann Rheum Dis. 2019;78(2):270-7. <https://doi.org/10.1136/annrheumdis-2018-213882>.

2. Lokau J, Göttert S, Arnold P, Düsterhöft S, Massa López D, Grötzinger J, et al. The SNP rs4252548 (R112H) which is associated with reduced human height compromises the stability of IL-11. Biochimica et Biophysica Acta (BBA) - Molecular Cell Research. 2018;1865(3):496-506. <https://doi.org/10.1016/j.bbamcr.2017.12.003>.

3. Styrkarsdottir U, Lund SH, Thorleifsson G, Zink F, Stefansson OA, Sigurdsson JK, et al. Meta-analysis of Icelandic and UK data sets identifies missense variants in SMO, IL11, COL11A1 and 13 more new loci associated with osteoarthritis. Nature Genetics. 2018;50(12):1681-7. <https://doi.org/10.1038/s41588-018-0247-0>.

4. Boer CG, Yau MS, Rice SJ, Coutinho de Almeida R, Cheung K, Styrkarsdottir U, et al. Genome-wide association of phenotypes based on clustering patterns of hand osteoarthritis identify WNT9A as novel osteoarthritis gene. Ann Rheum Dis. 2020;80(3):367-75. <https://doi.org/10.1136/annrheumdis-2020-217834>.

5. den Hollander W, Boer CG, Hart DJ, Yau MS, Ramos YFM, Metrustry S, et al. Genome-wide association and functional studies identify a role for matrix Gla protein in osteoarthritis of the hand. Ann Rheum Dis. 2017;76(12):2046-53. <https://doi.org/10.1136/annrheumdis-2017-211214>.

6. Castano-Betancourt MC, Evans DS, Ramos YF, Boer CG, Metrustry S, Liu Y, et al. Novel Genetic Variants for Cartilage Thickness and Hip Osteoarthritis. PLoS Genet. 2016;12(10):e1006260. <https://doi.org/10.1371/journal.pgen.1006260>.

7. Cui G, Liu D, Wei R, Wu J, Liu R, Wang K. Association of rs2862851 in TGFA Gene with Peripheral TGFA Levels and the Severity of Knee Osteoarthritis in the Han Chinese Population. Genet Test Mol Biomarkers. 2020;24(12):771-6. <https://doi.org/10.1089/gtmb.2020.0119>.
