# Supplementary figures and images for: A human in vitro 3D neo-cartilage model to explore the response of OA risk genes to hyper-physiological mechanical stress
Source: Osteoarthr Cartil Open. 2021 Dec 25;4(1):100231. doi: 10.1016/j.ocarto.2021.100231 (PMC9718246; doi:10.1016/j.ocarto.2021.100231)

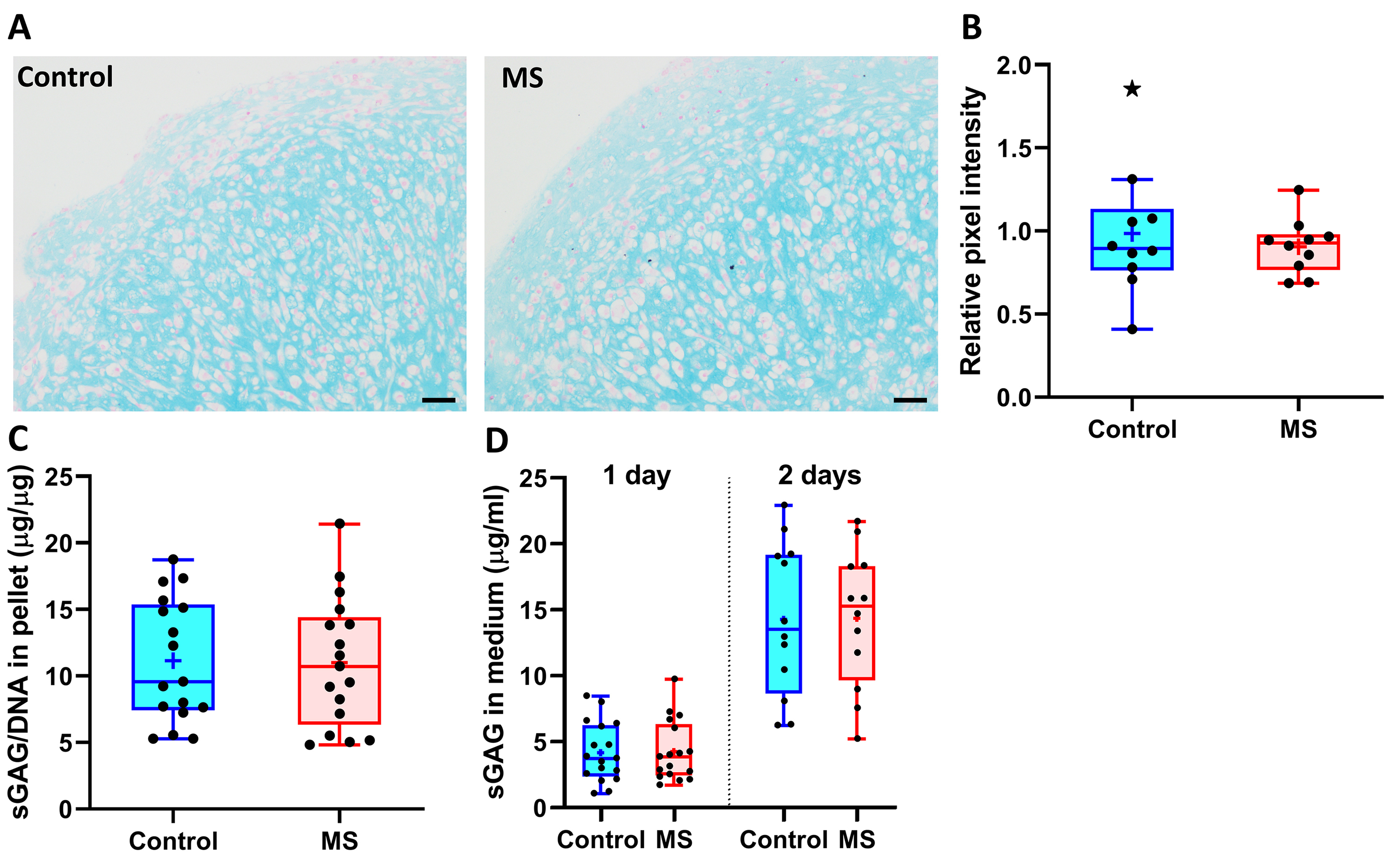

Supplement: figs1Fig. S1 — Assessment of damage in the neo-cartilage after the first day of 20% MS. (A-B) Representative images of Alcian Blue staining and quantification of Alcian Blue intensity calculated using Fiji-ImageJ. Scale bar = 50 μm. (C) sGAG content in the neo-cartilage as determined by DMMB. (D) sGAG concentrations in the medium after the first and the second day of MS as determined by DMMB. Data are presented in a boxplot depicting the median, lower and upper quartiles. Mean is depicted as +, each dot represents a single donor, and ∗ represents a statistical outlier. P-values of mean differences in Alcian Blue intensities (N = 10 donors) and sGAG levels (N = 17 donors) between controls and MS neo-cartilage were estimated by generalized estimating equations (GEE). [file figs1.jpg]
